# Comparison of class 2 transposable elements at superfamily resolution reveals conserved and distinct features in cereal grass genomes

**DOI:** 10.1186/1471-2164-14-71

**Published:** 2013-01-31

**Authors:** Yujun Han, Shanshan Qin, Susan R Wessler

**Affiliations:** 1 Department of Plant Biology, University of Georgia, Athens, GA 30602, USA; 2 Department of Botany and Plant Sciences, University of California, Riverside, CA 92521, USA

**Keywords:** Genome comparison, Plant genomes, Genome evolution, Class2 transposable elements, Features, Grass genomes

## Abstract

**Background:**

Class 2 transposable elements (TEs) are the predominant elements in and around plant genes where they generate significant allelic diversity. Using the complete sequences of four grasses, we have performed a novel comparative analysis of class 2 TEs. To ensure consistent comparative analyses, we re-annotated class 2 TEs in *Brachypodium distachyon*, *Oryza sativa* (rice), *Sorghum bicolor* and *Zea mays* and assigned them to one of the five cut-and-paste superfamilies found in plant genomes (*Tc1/mariner, PIF/Harbinger, hAT, Mutator, CACTA*). We have focused on noncoding elements because of their abundance, and compared superfamily copy number, size and genomic distribution as well as correlation with the level of nearby gene expression.

**Results:**

Our comparison revealed both unique and conserved features. First, the average length or size distribution of elements in each superfamily is largely conserved, with the shortest always being *Tc1/mariner* elements, followed by *PIF/Harbinger*, *hAT, Mutator* and *CACTA*. This order also holds for the ratio of the copy numbers of noncoding to coding elements. Second, with the exception of *CACTAs,* noncoding TEs are enriched within and flanking genes, where they display conserved distribution patterns, having the highest peak in the promoter region. Finally, our analysis of microarray data revealed that genes associated with *Tc1/mariner* and *PIF/Harbinger* noncoding elements have significantly higher expression levels than genes without class 2 TEs. In contrast, genes with *CACTA* elements have significantly lower expression than genes without class 2 TEs.

**Conclusions:**

We have achieved the most comprehensive annotation of class 2 TEs to date in these four grass genomes. Comparative analysis of this robust dataset led to the identification of several previously unknown features of each superfamily related to copy number, element size, genomic distribution and correlation with the expression levels of nearby genes. These results highlight the importance of distinguishing TE superfamilies when assessing their impact on gene and genome evolution.

## Background

Transposable elements (TEs) are DNA fragments that can move from one genomic location to another by a process called transposition. They are the largest component of most multicellular organism genomes, often exceeding 50% of content [1,2]. TEs are divided into two classes based on the nature of their transposition intermediate: class 1 (RNA) retrotransposons and class 2 DNA transposons. If we think of the genomes of higher plants as ecosystems, then each TE class occupies a characteristic niche. Although class 1 TEs are largely intergenic, most class 2 TEs are preferentially found in and around genes. As such, class 1 elements contribute more significantly to plant genome size differences whereas class 2 elements are frequently involved in generating allelic diversity [3].

Transposition of class 2 elements, which are the focus of this study, requires the enzyme transposase that binds to the elements’ terminal inverted repeat (TIR) and catalyzes both excision and integration into a new site. Class 2 elements are classified into superfamilies based on the relatedness of the transposase and on shared structural features including the TIR sequence and the length of the target site duplication (TSD) that flanks the TIR and is generated during integration. Only five of the seventeen superfamilies characterized to date have been found in plant genomes (*CACTA*, *Mutator*, *PIF/Harbinger*, *hAT*, *Tc1/mariner*) [4,5]. While helitrons were considered to be the sixth class 2 superfamily in plants [6], we do not include them here because they have different structures and transposition mechanisms from cut-and-paste elements. Each superfamily contains autonomous and nonautonomous elements. Autonomous elements encode the transposase necessary for their own movement and the movement of nonautonomous superfamily members, which lack functional transposase genes.

The most numerous class 2 elements in characterized plant genomes and in several animal species are miniature inverted-repeat transposable elements (MITEs). MITEs are nonautonomous TEs that are characterized by short length (most < 600bp), high sequence similarity, and a potential for very high copy number (hundreds or thousands) [7-9]. Despite lacking coding sequences, MITEs can be classified into known superfamilies based on related TIR sequence and TSD length. To date, the majority of reported MITEs are either *Stowaway* or *Tourist* elements that belong to the *Tc1/mariner* and *PIF/Harbinger* superfamilies, respectively [10-13]. MITEs belonging to the *hAT* and the *Mutator* superfamilies have also been reported [14-17].

Although TE superfamilies can be readily distinguished by shared sequences and structural features (such as TIRs and TSDs), TEs are usually lumped together when their relationship with gene and genome evolution is analyzed [18,19]. A determination of potentially unique and conserved features of each superfamily would require their systematic comparison across species. The availability of complete genome sequences from four grass species, *Brachypodium distachyon* (250Mb) [20], *Oryza sativa* (rice)(340Mb) [21], *Sorghum bicolor* (750Mb) [22], and *Zea mays* (2500Mb) [1] has facilitated such a novel comparative analysis. These four grass species were chosen for several reasons. First, the genomes have been annotated to high quality and some gene expression data is available. Although TEs have also been annotated in these genomes, we performed a systematic re-annotation to permit a consistent comparative analysis and to classify noncoding elements into superfamilies. With only five of the ~20 class 2 cut-and-paste superfamilies identified in eukaryotes, it is more likely that most TEs in the genomes can be assigned. In contrast, with over 17 superfamilies, it is very difficult to classify *Aedes aegypti* TEs into superfamilies unambiguously [2]. In this study, we identified and classified over 450,000 class 2 elements. Finally, the genes in these four species are largely syntenic despite a 10-fold difference in genome size. Given that most class 2 elements are known to insert into or near genes, we were particularly interested in comparing each TE superfamily across species to determine what features are conserved and what features may be influenced by the host.

## Results

### TE annotation and definitions

**Figure 1:**
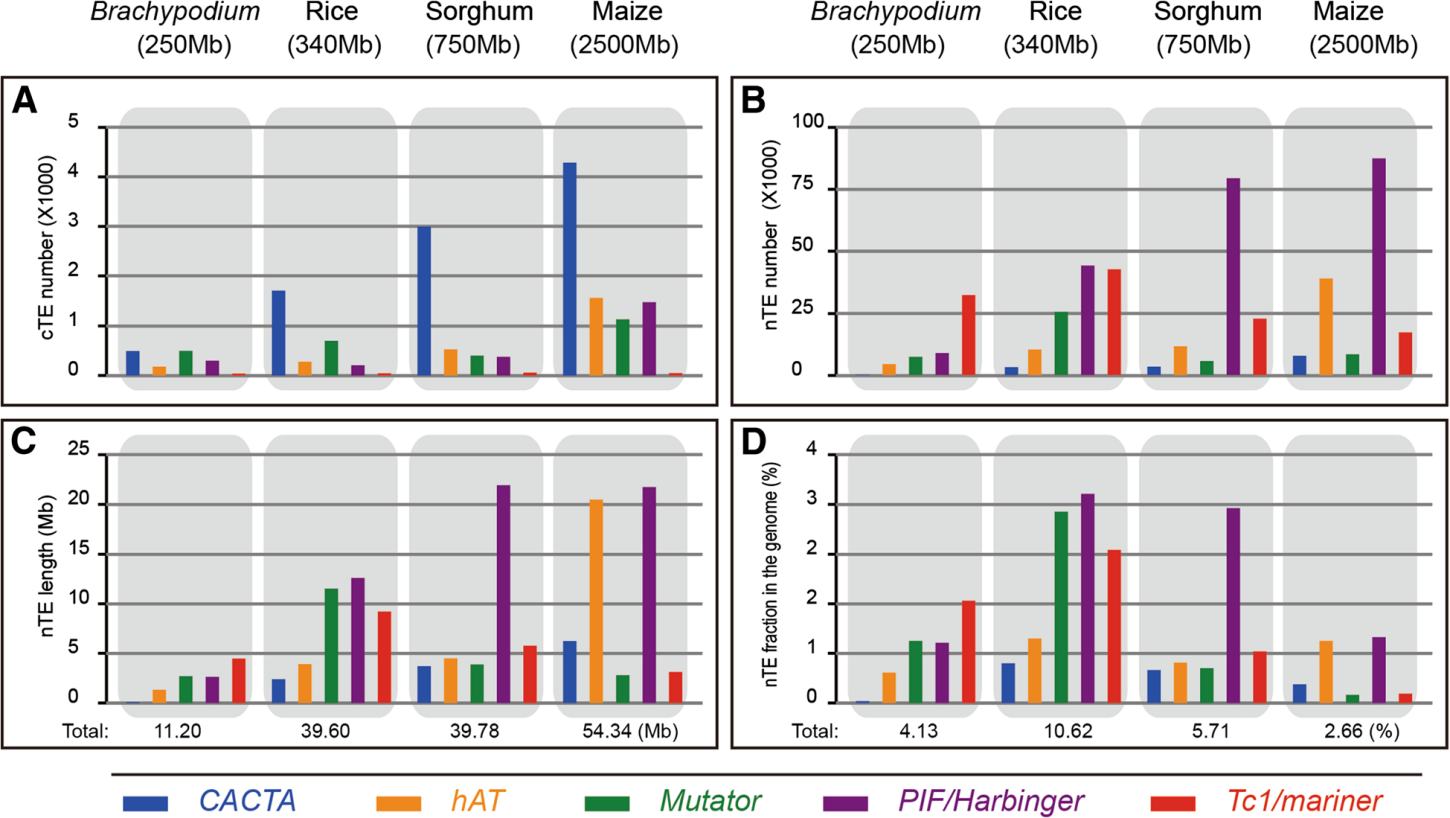
**TE annotation results in four grass genomes.** Each superfamily is represented by a specific color (*CACTA:* blue, *hAT:* dark orange, *Mutator:* green, *PIF/Harbinger:* purple and *Tc1/mariner:* red). **A**) The number of transposase genes identified by TARGeT. **B**) Copy number of nTEs. A copy that has only one end was counted as 0.5. **C**) Total length of nTEs. **D**) Percentages of nTEs in the genomes.

Class 2 TEs from the five superfamilies found in plants were annotated in four grass genomes and the results are summarized in Figure 1 and Additional file 1. Coding elements contain all or part of the transposase gene from one of the five superfamilies. Their copy numbers were estimated from the number of conserved transposase domains identified by TARGeT (Figure 1A and Additional file 1) [22]. We call them coding elements rather than the more conventional autonomous elements because the latter term implies functional activity, and manual curation of a subset of these elements indicates that most contain inactivating mutations. To save words, coding TEs are denoted with a lowercase “c” followed by the superfamily name (e.g. the generics cTE as in *cCACTA*). Similarly, noncoding elements are referred to as nTEs. In this study nTEs were discovered by MITE-Hunter, a structure based TE identification tool that has as its output consensus sequences that represent nTE families. By default MITE-Hunter identifies nTEs shorter than 2kb, which encompasses the majority of nTEs including MITEs [23].

To obtain the copy number, length and position of nTEs in each genome, consensus sequences were used as queries for searches using RepeatMasker. From the RepeatMasker output, double-ended elements and single-ended elements were identified and analyzed separately (see *Methods* for details). The total copy number of a nTE consensus sequence was calculated by adding the double-ended copy number and half of the single-ended copy number. Furthermore, nTEs were characterized as either MITE or non-MITE where MITEs are defined as shorter than 600bp with at least 25 double-ended copies or 10 nearly identical copies (identity > = 99%) (Additional file 1). The total copy number and length of nTEs (Figure 1B and C) as well as the percentage of each superfamily in the four genomes (Figure 1D) was determined. While these data are combined in Figure 1 and Additional file 1, the complete dataset is available at http://target.iplantcollaborative.org.

### Copy numbers of class 2 elements in four grass genomes

Except for the *Tc1/mariner* superfamily, the copy numbers of cTEs differ dramatically among the four grass genomes (Figure 1A and Additional file 1). *cCACTAs* are the most numerous in rice (340Mb), sorghum (750Mb) and maize (2500Mb) and are the second most numerous (after *Mutator*) in the *Brachypodium* genome (250Mb). Furthermore, *cCACTAs* increase in copy number with increasing genome size (491 to 1705 to 2996 to 4288). *chATs* also increase with genome size but their numbers are significantly lower than *cCACTAs* (176, 277, 526, 1556). Coding members of the *Mutator* and *PIF/Harbinger* superfamilies show little copy number variation in *Brachypodium*, rice and sorghum but show a marked increase in the larger maize genome. Interestingly, the coding members of the *Tc1/mariner* superfamily have by far the lowest copy numbers in the four genomes and show no correlation with genome size (36, 45, 57, 47).

Despite the dramatic copy number differences across species, the copy number ratio of nTEs to cTEs is a conserved feature of each superfamily (Additional file 1). For each genome, the ratio is lowest for the *CACTA* superfamily (1.27 average ratio), which has the most cTEs and the fewest nTEs. After *CACTA*, the next lowest ratio in all genomes is for *Mutator* (18.14) followed by *hAT* (27.16), *PIF/Harbinger* (128.53) and *Tc1/mariner* (647.25). The high ratios for *PIF/Harbinger* and *Tc1/mariner* reflect the success of MITEs from these superfamilies, called *Tourist* and *Stowaway*, respectively [7,8]. These results indicate that the mechanisms underlying the generation and success of nTEs are both conserved and distinctive for each superfamily.

### Length is a conserved feature of the nTEs in a superfamily

**Figure 2:**
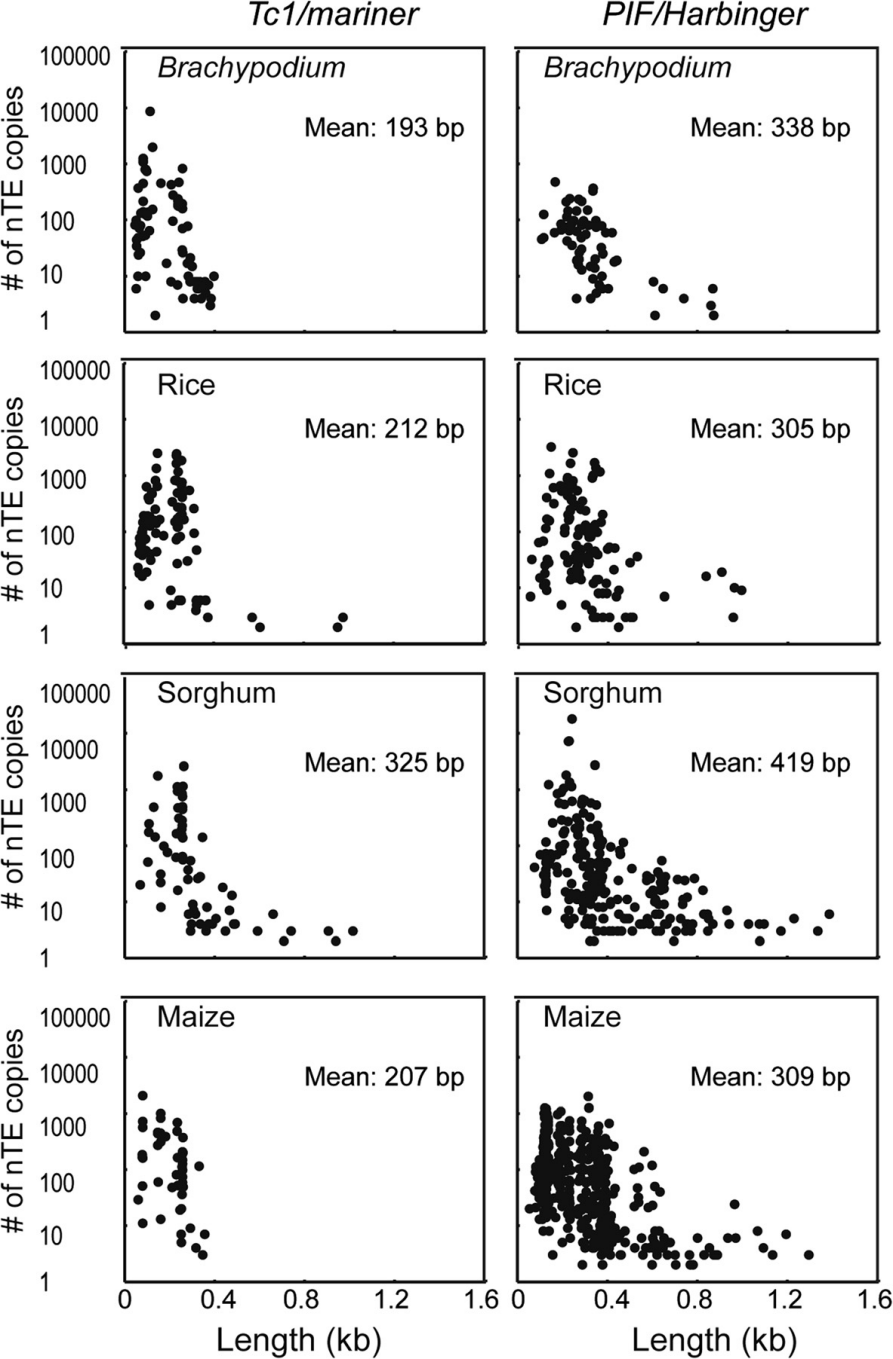
**The double-ended copy number and length of the consensus nTEs of *Tc1/mariner and PIF/Harbinger *in *Brachypodium*, rice, sorghum and maize.** The y-axis is in log10 scale.

Figure 2 is a visual representation of the size and copy number of the nTEs from *Tc1/mariner* and *PIF/Harbinger* in the four genomes (the results of all superfamilies except *CACTA* are in Additional file 2). These data were generated by plotting the double-ended copy numbers of each consensus nTE against the length of the consensus sequence. Of note is the similarity of the patterns for a particular nTE superfamily in all genomes. In addition, with only one exception (*nhAT* and *nMutator* in maize), the order of the mean lengths of the nTEs is the same in all genomes with *nTc1/mariner* < *nPIF/Harbinger* < *nhAT* < *nMutator*.

### All nTEs except *nCACTAs* are enriched near genes

Prior studies showed that most plant class 2 TEs have a preference for insertion into or near genes [3,24,25]. Recently, the determination of over 800 insertions of the active MITE *mPing* near rice genes identified a preference for insertion of this member of the *PIF/Harbinger* superfamily within 1kb of the transcription start site (TSS) and 1kb downstream of the transcription stop site (TTS) [26]. Here our annotation results have been used to determine whether nTEs from other superfamilies show similar enrichment and, if so, whether this feature is conserved in all genomes.

**Figure 3:**
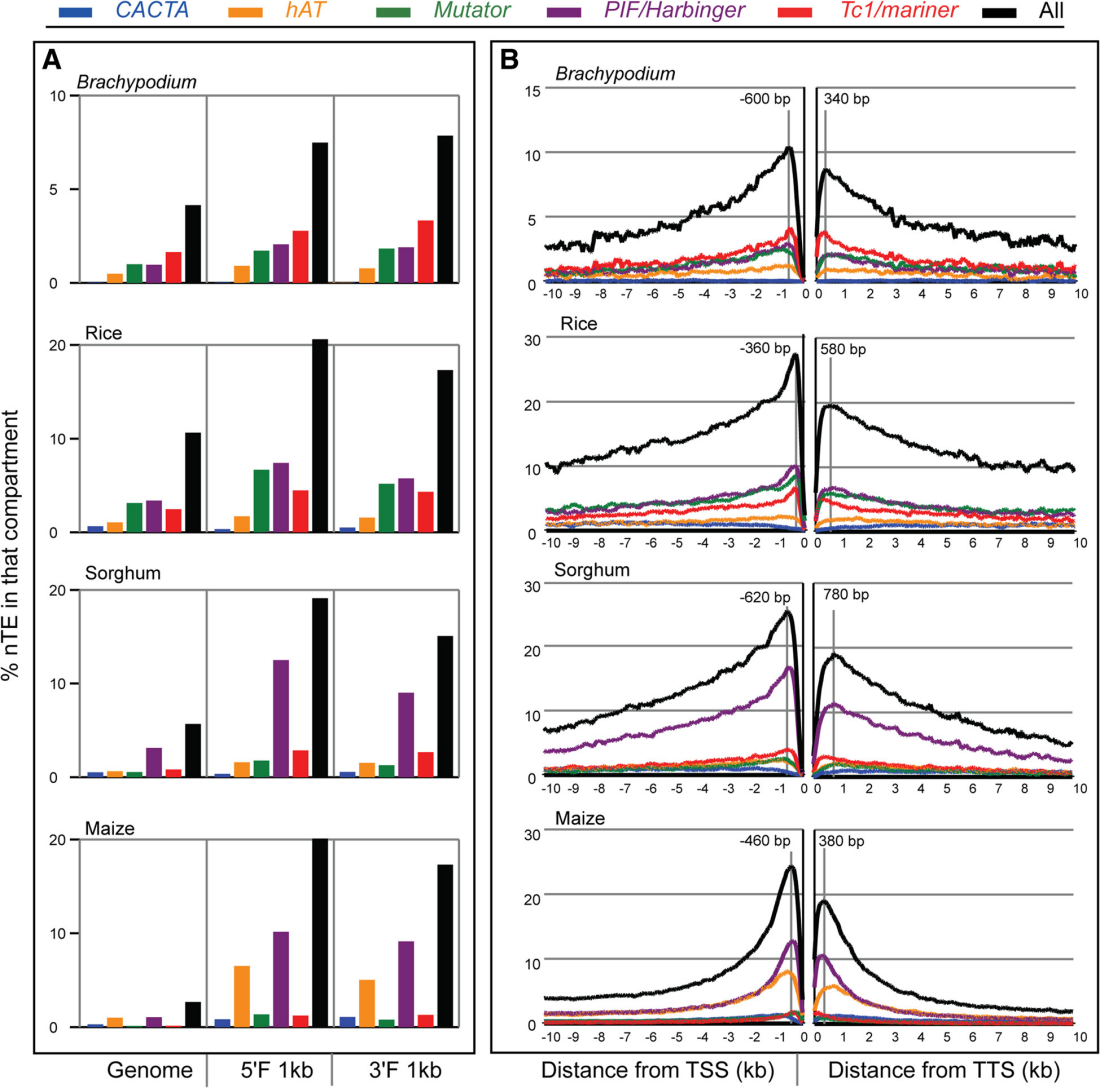
**The nTE distribution in four grass genomes. A**) The length percentage of nTEs in the whole genome, 1kb 5^′^ gene flanking regions (5^′^F), and 1kb 3^′^ gene flanking regions (3^′^F) of *Brachypodium*, rice, sorghum and maize. **B**) High resolution distribution of nTE frequency around the genes of *Brachypodium*, rice, sorghum and maize. The highest peaks of nTE frequency are marked by vertical gray lines and the distance from the peak to the TSS (transcription start site) or TTS (transcription stop site) is shown above the lines. Colors of superfamilies are the same as in Figure 1.

To this end we first calculated the average percentages of class 2 nTEs in the whole genome as well as in the 5^′^ and 3^′^ flanking regions around genes. These data are presented diagrammatically in Figure 3A and in detail in Additional file 3 (this file also contains the distributions of all nTEs in the exons and introns, which is not discussed further). Taken together these data indicate that the nTEs from all superfamilies except *CACTA* are enriched in the 1kb compartments flanking the 5^′^ and 3^′^ ends of coding regions. The only exception is in maize where there are more *nCACTAs* near genes than in other areas of the maize genome, which may reflect the overwhelming number of class 1 LTR elements that are enriched in intergenic regions (composing 74.6% of the maize genome [1]) [3]. The extent of enrichment is especially pronounced in rice, sorghum and maize where about 20% of the sequences in these regions are derived from class 2 nTE sequences (Figure 3A and Additional file 3).

To characterize the distribution of nTEs at higher resolution and in regions more distant from the TSS and TTS, we calculated nTE percentages in continuous 10bp windows extending 10kb upstream and 10kb downstream of coding regions. The distributions of all nTEs in these regions are similar as evidenced by the black curves in Figure 3B, although the percentage of elements around the genes of *Brachypodium* is about half that of the other three species. After the 5^′^ and 3^′^ flanking regions, intron nTEs are the most abundant across all the superfamilies and species. However, unlike the patterns observed in the gene flanking regions, nTE frequencies are highest in the middle of introns and drop gradually toward the splice sites (see Additional file 4).

### nTEs differ in their association with genes of high or low expression

The abundance of class 2 nTEs in genic regions prompted us to examine whether the presence of a particular superfamily member near genes correlates with increased or reduced expression levels. This analysis was performed in rice and maize where annotation is of high quality and gene expression data is available. Genes harboring nTEs from members of the same TE superfamily were grouped and their expression levels were compared with a control group of genes without class 2 nTEs. Extensive comparisons were performed using rice microarray data from different experiments, tissues and platforms. A more limited comparison was also made with available maize expression data (see *Methods* for details).

**Figure 4:**
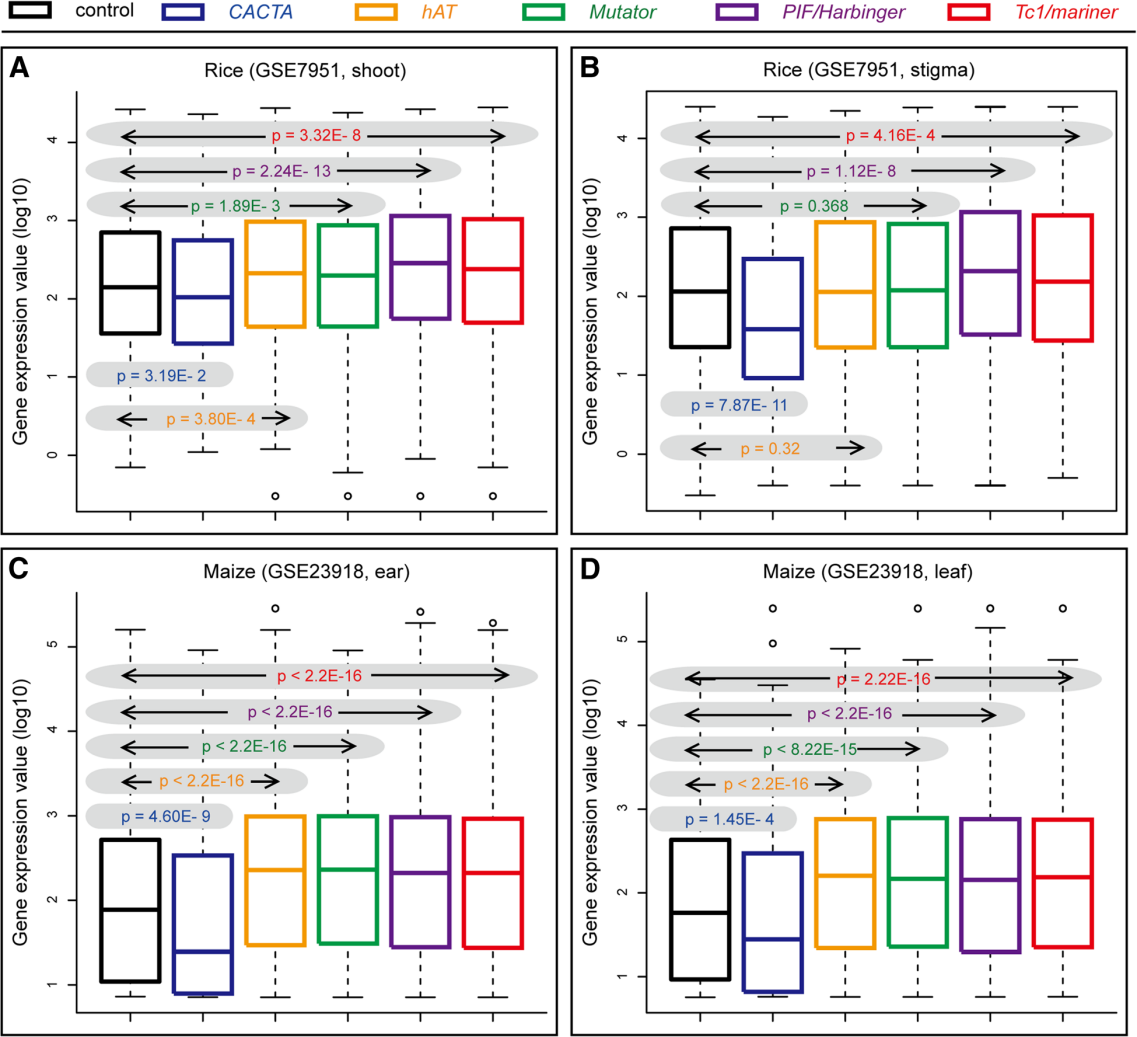
**Comparison between the expression levels of genes with class 2 TEs and without class 2 TEs, in rice root (A) and stigma (B), maize ear (C) and leaf (D). The y-axis is log10 gene expression values.** P-values of pairwise comparison are shown in shadow areas. Colors of superfamilies are the same as in Figure 1.

In all microarray data analyzed in rice and maize, genes with *nPIF/Harbinger* and *nTc1/mariner* elements have significantly higher expression levels than genes in the control group. In contrast, the expression of both rice and maize genes with *nCACTAs* is significantly lower than the control dataset (Figure 4 and Additional file 5). Although expression levels for maize genes associated with *nhATs* and *nMutators* are significantly higher than the controls in RNA samples from two tissues (Figure 4C and D), no clear picture emerged when rice samples were analyzed (Figure 4A and B, Additional file 5 GSE23918).

To confirm these findings, we analyzed publically available RNA-seq data from rice and obtained very similar results (see Additional file 6). Specifically, genes with class 2 superfamily nTEs except for *nCACTAs,* showed significantly higher expression levels than controls. The expression levels of genes with *nCACTAs* are lower than controls, but these data are not statistically significant (p-value = 0.0608), which can be explained by the small sample size and limited sequencing reads to discern genes with low expression.

## Discussion

The major focus of this study was to generate comprehensive and accurate class 2 TE data for comparative analyses. To this end we utilized TARGeT and MITE-Hunter, two programs that have proved efficient at detecting cTEs and nTEs, respectively [22,23]. In our analyses we separated nTEs from cTEs, classified them into superfamilies, and further identified MITEs among the nTEs. This protocol was necessary because nTEs and cTEs have distinct features. For example, coding *Tc1/mariners* have only about 50 copies in each of the analyzed genome but there are two orders of magnitude more n*Tc1/mariners* (mainly *Stowaways*) (Figure 1 and Additional file 1). The dramatic amplification potential of small nTEs, in particular MITEs, is also very different across superfamilies. Extensive manual curation was performed for each nTE consensus sequence to ensure the accuracy of TE discovery and classification. In this way, we achieved the most comprehensive annotation of class 2 cut-and-paste TEs to date in these four grass genomes. For example, we found several-fold more *Stowaway* and *Tourist* elements than a previous annotation of these elements in rice, sorghum and maize [22]. Comparative analysis of this robust dataset led to the identification of several previously unknown features related to copy number, element size, genomic distribution and correlation with the expression level of nearby genes.

The *CACTA* superfamily is the outlier in all comparisons. Among the superfamilies analyzed in this study, *CACTA* has the fewest number of nTEs and the greatest number of cTEs (Figure 1, Additional file 1). This paucity of *nCACTAs* suggests that this superfamily generates fewer short elements than the others. Further, in three of the four genomes analyzed, *CACTAs* are enriched in intergenic regions where their copy numbers increase proportionally with genome size (see Figure 1). Finally, in at least two grass species (maize and rice) the presence of *nCACTAs* in or near genes has a negative correlation with transcription (Figure 4). Taken together, these data suggest that *CACTA* elements have either evolved a genic region insensitive/avoidance strategy or are removed from genic regions by selection.

The other four class 2 TE superfamilies also have distinctive features that are conserved in all genomes analyzed. For example, the ratio of the number of nTEs to cTEs is the highest for the *Tc1/mariner* superfamily and next highest for *PIF/Harbinger* followed by *hAT*, *Mutator* and *CACTA* (Figure 1). In this same order, the average length of nTEs also increases (Figure 2 and Additional file 2) suggesting that there is a range of lengths that is optimal for the transposition and amplification of each superfamily.

With regard to the distribution of elements in the four superfamilies, we have extended an observation originally made in rice [27] and show that except for *nCACTAs* nTEs are enriched at gene borders (Figure 3). Specifically, nTEs are most abundant at the 5^′^ gene border, and also enriched but less so near the 3^′^ border (Figure 3 and Additional file 3). This result, however, differs from a recent report in rice [18]. For the *PIF/Harbinger* superfamily, enrichment of the active MITE *mPing* was shown previously to result from its preference for insertion into gene proximal regions [26]. Although our data are descriptive and as such cannot distinguish between an insertion preference or winnowing by selection, the strikingly similar patterns around grass genes suggests a preference. A similar insertion preference was observed for *Hermes*, an active member of the *hAT* superfamily from the housefly *Musca domestica*[28]. Characterization of almost two hundred thousand insertion sites in a *Saccharomyces cerevisiae* transposition assay revealed a marked preference for nucleosome free regions (NFRs) around genes, presumably because of their accessibility. Given that NFRs have also been found near the 5^′^ ends of plant genes [29] it is possible that their distribution underlies the pattern of nTE insertion sites from four of the five superfamilies in plants.

The dramatic enrichment of class 2 nTEs around genes especially in promoters prompted us to analyze the correlation between these elements and nearby gene expression levels using microarray data. Genes with *PIF/Harbinger* and *Tc1/mariner* elements, which are the two superfamilies that generate the majority of MITEs, showed significantly higher expression values (Figure 4, Additional files 5 and 6). Furthermore, genes with *hAT* and *Mutators* showed higher expression levels in maize but not in all rice tissues. In contrast, as discussed above, genes with *CACTA* elements were associated with lower gene expression.

## Conclusions

These results indicate that superfamily identity needs to be considered when analyzing the correlation between TEs and the expression of nearby genes. This conclusion may explain discrepancies between our results and those of prior studies. For example, Hollister et al. and Lu et al. reported negative association between TEs and nearby genes in Arabidopsis [30] and rice [18], respectively. Based on our results, a possible explanation is that in both of these studies TEs were not separated into superfamilies for the analysis. Grouping of TEs in this way, without regard for superfamily identity, could mask the unique behavior of individual superfamilies. Finally, as our data and prior studies have shown, nTEs, especially MITEs, are abundant near genes [27]. It would be difficult to explain this distribution if the impact of these elements were largely negative.

## Methods

### Dataset

Genomic sequences and gene annotation results (version 1.0) of *Brachypodium distachyon* were downloaded from http://www.brachypodium.org[20]. Rice genome (build 5) was downloaded from http://rgp.dna.affrc.go.jp/, and gene annotation file (RAP3.gff3) was from http://rapdb.dna.affrc.go.jp[21,31]. Sorghum genome (Sorbi1) and annotations (Sbi1_4.gff) were downloaded from http://genome.jgi-psf.org/Sorbi1/Sorbi1.home.html[22]. Maize genome and annotations (version 4a53) were from http://www.maizesequence.org[1]. Microarray data files were downloaded from the Gene Expression Omnibus (GEO) database [32]. Rice RNA-seq data were downloaded from EBI (ERR008651, ERR008652, ERR008657, ERR008658, ERR008663 and ERR008664).

### Discovery and classification of class 2 TEs

Class 2 TEs were discovered from genomic sequences as follows. Conserved transposase gene regions of putative autonomous TEs were identified using TARGeT [22]. Query sequences for TARGeT were curated from the conserved regions of known plant transposase sequences that were downloaded from Repbase [33]. Each transposase gene discovered in this way was considered to represent a single coding element (as listed in Additional file 1).

Noncoding elements were discovered using MITE-Hunter, which is a tool designed to specifically search for small noncoding class 2 TEs from genomic sequences [23]. MITE-Hunter outputs were manually checked using the approach described previously [23]. Qualified TE consensus sequences were classified into superfamilies based on their TIR sequences and TSD length using the following rules. Elements with TIRs starting with CACTA/G and with 3bp TSDs were identified as *CACTA* TEs, with 8bp TSDs as *hAT* TEs, with 9 or 10bp TSDs as *Mutator* TEs, with 2bp TSDs that are TA as *Tc1/mariner* TEs and with 3bp TSDs that are TAA or TTA as *PIF/Harbinger* TEs. Nested TEs were removed and low complexity sequences were masked before homology searches by RepeatMasker described below.

Because one TE copy may mutate into several fragments, the number of total fragments is higher than the total copy number of TEs. We counted the nTE copy number using an approach similar to one introduced previously (see Additional file 1, the 6^th^ column) [1]. Total length and copy numbers of class 2 noncoding TEs were determined using RepeatMasker as follows. Curated MITE-Hunter outputs were first masked by mdust (http://compbio.dfci.harvard.edu/tgi/software/) to filter low complexity regions and were used as library files for RepeatMaker (version 3.26, http://www.repeatmasker.org) to mask the genomic sequences with “–nolow” and “–no_is” parameters. Because the positions of some TE copies overlap in the RepeatMasker output, to avoid counting a TE region twice, the original RepeatMasker output was first filtered and then the length and copy number of TEs were counted, which was done by a Perl script introduced in detail as follows. In the RepeatMasker output, if two consecutive TE copies overlapped, then the starting position of the second one was adjusted so it was right after the stop position of the first one. If a short TE copy was within a longer one, the shorter one was filtered. If a TE copy identified by RepeatMasker was missing less than 20bp from both ends of the query nTE consensus sequence, it was considered to be a double-ended copy. If a TE copy had only one end that was missing less than 20bp, it was counted as half of a copy. Other TE copies in the RepeatMasker output were considered as fragments that were not counted in the copy numbers. A nTE was considered to be a MITE if it was less than 600bp and had at least 25 double-ended copies, or 10 double-ended copies with an identity > = 99%.

### Calculation of TE distribution within and around genes

Several Perl scripts were written to acquire the genomic positions of TE regions and genes and to render figures of the distribution and proportion of TE sequences in and around genes. Positions of TE sequences were retrieved from processed RepeatMasker outputs. Positions of genes were retrieved from gene annotation files as follows. First, annotation files were checked to filter genes whose exons have contradicted directions. Genes inside other genes were also filtered. Second, for each gene, positions of different compartments were retrieved including 5^′^ flanking regions, 5^′^ UTR exons, 5^′^ UTR introns, exons, introns, 3^′^ UTR exons, 3^′^UTR introns and 3^′^ flanking regions. Due to alternative splicing, one gene may have several sets of annotated compartments. In such cases, a combined gene model was generated using the following rules. If there was conflicted annotation information between two models, highest priority went to exons in the coding regions, with exons in the UTR next and introns with lowest priority. For example, a region annotated as an exon in one gene model but an intron in another was considered as an exon only.

For each type of gene compartment, an average percentage was calculated by dividing the total length of TE sequences within the gene compartment by the total length of the gene compartment. In the 5^′^F and the 3^′^F of all genes, TE sequence proportions were also calculated along each nucleotide position.

### Analyses of microarray expression data

Gene expression values were acquired from microarray data in the GEO database. Rice data was from four sample series from two platforms: GPL6864 (Rice 44K, product of Agilent) and GPL2025 (Affymetrix Rice Genome Array). GPL6864 was designed based on RAP annotation results but GPL2025 was based on TIGR annotations. Because we used RAP annotations in this study, to use GPL2025 microarray data, we performed sequence comparisons between each GPL2025 probe sequence with RAP cDNA sequences. Maize expression data was from platform GPL10837 (Maize Pioneer Hi-Bred 105K mRNA Microarray). Because correlations between probe IDs and gene names in the gene annotation file were not known, as with the rice data, we performed comparisons between maize probes and maize cDNA sequences. For all microarray platforms, only genes whose probes had 100% identity to cDNA sequences were used. If a probe matched more than one gene its expression data was not used. If a gene had more than one probe, an average value was calculated.

### Analyses of RNA-seq data

Reads were mapped to the rice reference genome using BWA [34]. PCR redundant reads were identified and filtered using picard (http://picard.sourceforge.net). Reads mapped within 80bp to the microarray probes were counted to represent the expression level of each gene.

### Statistical tests of the association between TEs and genes

Brunner-Munzel Test (Generalized Wilcoxon Test) was carried out using R package (version 2.12.0) to compare the expression levels between each TE-containing gene group with the control gene group. Genes (including 1kb flanking regions) that contain the same superfamily of class 2 nTEs were put into the same group. Genes that did not have any class 2 nTEs were put into the control group. Only genes with at least 1kb flanking region on either side were used in this analysis.

## Competing interests

The authors declare that they have no competing interests.

## Authors’ contributions

SRW and YH designed the experiments and wrote the manuscript. YH developed programs and performed the primary data analyses. SQ participated the analyses and performed the statistical tests. All authors read and approved the final manuscript.

## Supplementary Material

Additional file 1 Summary of Class 2 transposable elements from five superfamilies in four sequenced grass genomes. Click here for file

Additional file 2 **The double-ended copy number and length of the consensus nTEs of *Tc1/mariner*, *PIF/Harbinger*, *hAT*and *Mutator*in *Brachypodium*, rice, sorghum and maize.** The y-axis is in log10 scale. Click here for file

Additional file 3 Total length of nTEs from each superfamily genome-wide, intergenic and in four genic compartments. Click here for file

Additional file 4 **Distribution of nTE sequence frequency within introns of *Brachypodium *(A), rice (B), sorghum (C) and maize (D).** The left and right starting points represent the 5^′^ and 3^′^ borders of introns, respectively. Colors of superfamilies are the same as in Figure 1. Click here for file

Additional file 5 Comparison of the expression of genes associated with different class 2 TE superfamily members vs. genes without class 2 TEs (control) (using microarray data). Click here for file

Additional file 6 Comparison of the expression of genes associated with different class 2 TE superfamily members vs. genes without class 2 TEs (control) (using RNA-seq data). Click here for file
