# Supplementary figures and images for: Comparison of class 2 transposable elements at superfamily resolution reveals conserved and distinct features in cereal grass genomes
Source: BMC Genomics. 2013 Jan 31;14:71. doi: 10.1186/1471-2164-14-71 (PMC3579700; doi:10.1186/1471-2164-14-71)

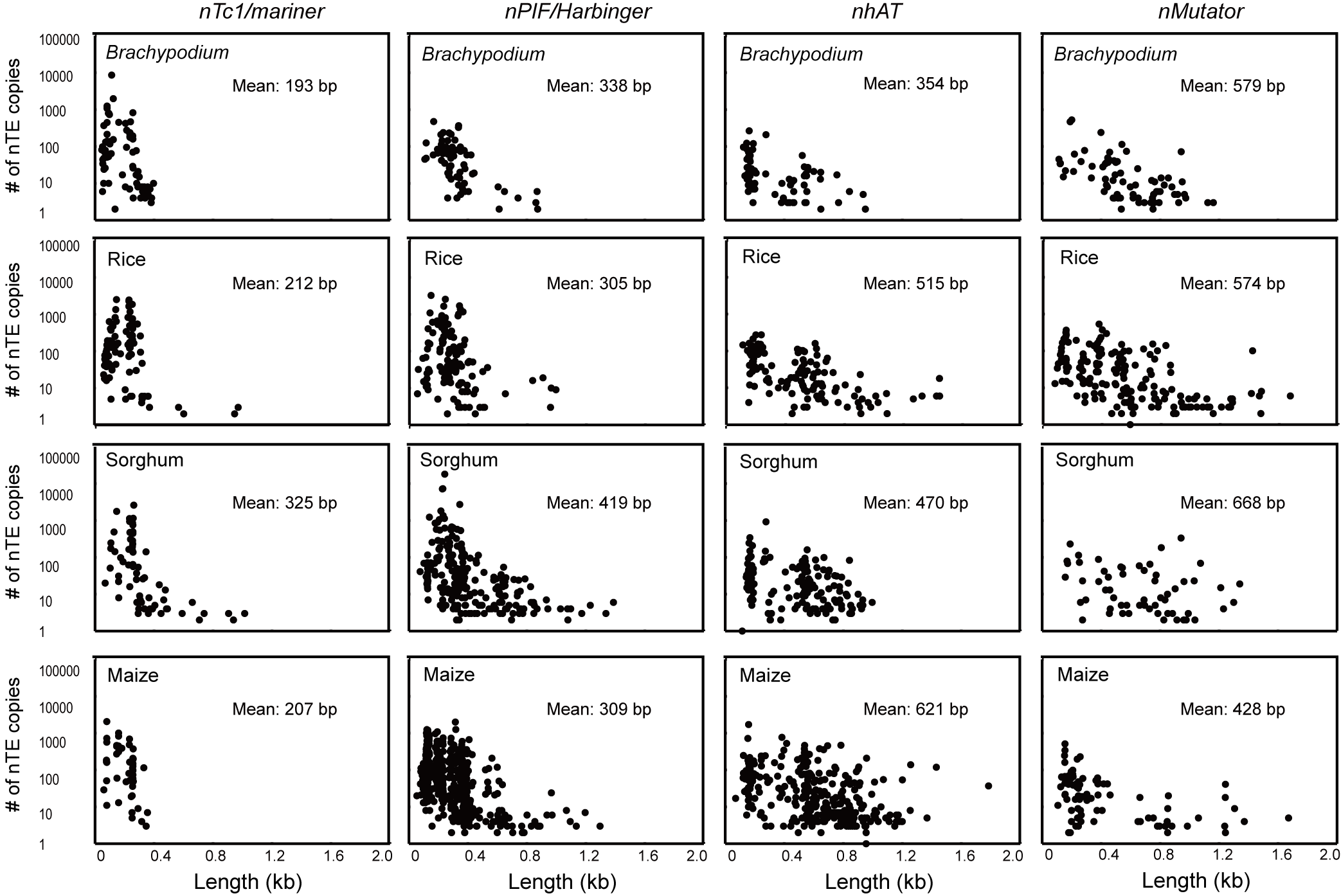

Supplement: Additional file 2 — The double-ended copy number and length of the consensus nTEs of Tc1/mariner, PIF/Harbinger, hATand Mutatorin Brachypodium, rice, sorghum and maize. The y-axis is in log10 scale. [file 1471-2164-14-71-S2.tiff]

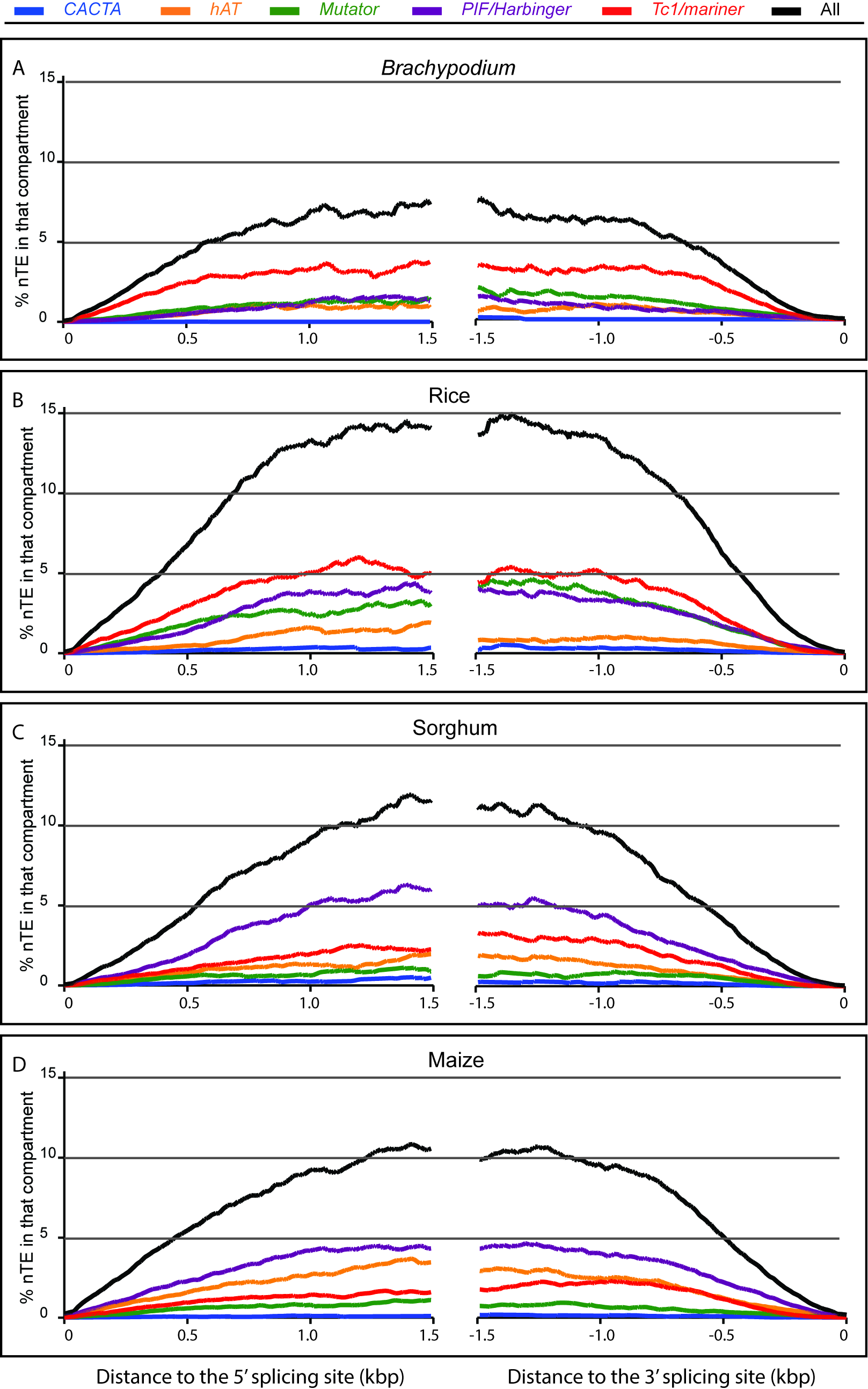

Supplement: Additional file 4 — Distribution of nTE sequence frequency within introns of Brachypodium (A), rice (B), sorghum (C) and maize (D). The left and right starting points represent the 5′ and 3′ borders of introns, respectively. Colors of superfamilies are the same as in Figure 1. [file 1471-2164-14-71-S4.tiff]
